# Impact of Action Observation Therapy on Circumduction Gait in Stroke Patients

**DOI:** 10.7759/cureus.110542

**Published:** 2026-06-09

**Authors:** Yukti K Agrawal, Suraj Kanase

**Affiliations:** 1 Department of Neurosciences, Krishna College of Physiotherapy, Krishna Vishwa Vidyapeeth (Deemed to Be University), Karad, IND

**Keywords:** gait training, hemiplegic gait, motor learning, neuroplasticity, stroke survivors

## Abstract

Background: Circumduction gait is a common compensatory movement pattern used by stroke patients, which significantly affects mobility and quality of life. Action observation therapy (AOT), an emerging rehabilitation approach based on the mirror neuron system, has shown promise in motor recovery. This study explores the impact of AOT on circumduction gait in poststroke individuals.

Objective: This study aims to evaluate the effectiveness of AOT in reducing circumduction gait and improving overall gait kinematics in stroke survivors.

Methods: A pre-post experimental study was conducted involving 30 stroke patients exhibiting circumduction gait. Participants were divided into an intervention group (n = 15) receiving AOT alongside conventional physiotherapy and a control group(n = 15) undergoing physiotherapy alone. The AOT group watched videos of normal gait patterns and actively attempted to imitate them. Gait parameters, including hip circumduction angle, stride length, and step width, were assessed pre- and postintervention using the footprint method and motion analysis (by Kinovea software; developed by Joan Charmant and Contributors, Kinovea Open Source Project, Bordeaux, France).

Results: Patients in the AOT group demonstrated significant improvements in gait symmetry, reduced circumduction angle, increased stride length, and reduced step width compared to the control group (p < 0.05). The findings suggest that AOT facilitates motor relearning and reduces compensatory gait deviations.

Conclusion: AOT is an effective adjunct to conventional stroke rehabilitation, aiding in correcting circumduction gait. Its implementation may enhance functional recovery and mobility in stroke patients, ultimately improving independence and quality of life. Further studies with larger sample sizes are recommended to validate these findings.

## Introduction

Hemiplegia involves impairment of one side of the body, which can present with the arm affected more than the leg, or with the leg affected as much as or more than the arm [1]. It is among the most common impairments following a stroke and significantly contributes to walking difficulties. A study conducted in 2023 in India reported an incidence rate of stroke ranging from 116 to 163 per 100,000 people. Approximately 66% of the Indian population resides in rural areas, and among these rural inhabitants, about 71% of stroke-related deaths have occurred, highlighting a disproportionately high number of fatalities [2].

The prevalence rates of stroke in rural India were found in studies that included individuals of all ages, ranging from 55 to 388.4 per 100,000 people [3]. The most common type of impairment was motor-related, with over 80% of patients experiencing some form of motor impairment [3]. This included facial palsy (50%), dysarthria (47%), limb weakness (30%), and ataxia (17%), with similar frequencies observed for weakness in both upper and lower limbs and between the right and left sides [3].

Around 66% of people who have had a stroke and had an initial loss of the ability to use one leg will be able to ambulate again either independently or with assistance [4]. The most commonly seen abnormal gait following a stroke is known as a circumduction gait or hemiplegic gait. This occurs when the hip and knee do not flex properly, causing the patient to swing their leg in an arc-like pattern to clear their foot.

Patients will generally walk very slowly, with great difficulty and only for short distances. Thus, the efficiency of their movement is poor. In addition, abnormal movement patterns are likely to create abnormal forces on the joints, increasing the likelihood of falling. Because of the immobility associated with a stroke, there will also be reductions in physical/mobility functions, along with reductions in overall quality of life and psychological well-being [4].

In conventional gait training, patients practice various functional locomotor skills, including 1) task-specific activities such as walking forward and side-stepping (for five minutes); 2) elevation exercises like step-ups, step-downs, lateral step-ups, and stair climbing; 3) community activities that involve walking on ramps, curves, and navigating around obstacles; and 4) quadriceps strengthening exercises [5]. While conventional gait training has proven effective, it often requires a longer recovery period. Therefore, additional interventions to improve circumduction gait are necessary.

Action observation therapy (AOT) is based on the activation of the mirror neuron system (MNS), a network of neurons that fire both when an individual performs an action and when they observe the same action being performed by another person. This shared neural activity facilitates motor learning, motor planning, and neuroplasticity, making AOT an effective intervention for neurological rehabilitation, particularly after stroke. In this method, patients observe specific actions, typically through video demonstrations, and then perform those same actions themselves. This process activates neural pathways associated with action observation and execution, facilitating motor learning and rehabilitation [6,7]. One advantage of AOT is that the observer simulates movement during observation, leading to more efficient learning than in general physical therapy. Additionally, AOT can strengthen the cortical network and activate specific regions of the cerebral cortex, even in patients with reduced physical function who have difficulty moving [7,8].

Circumduction gait is an alternative compensatory movement usually observed in individuals with stroke that affects their ability to ambulate efficiently, along with a greater risk of falling. Rehabilitative treatment usually fails to account for and treat any underlying motor impairment. Recent evidence suggests that using a method called AOT may improve motor recovery [6]. The objective of this research is to determine whether AOT can reduce circumduction gait and improve functional mobility compared with traditional physical therapy.

## Materials and methods

Design study and setting

This pre-post experimental study focused on hemiplegic patients exhibiting a circumduction gait. It was conducted in 2025 (from May 11, 2025, to May 5, 2026) at the Krishna College of Physiotherapy, Neurosciences OPD, under Krishna Vishwa Vidyapeeth College of Physiotherapy in Karad. Ethical clearance was obtained from the institutional committee.

Participants

The study population consisted of individuals with chronic hemiplegic stroke attending the Neurophysiotherapy Outpatient Department. A consecutive sampling technique was used, whereby all eligible patients presenting during the study period were screened according to the predefined inclusion and exclusion criteria until the required sample size was achieved. Participants aged 30-50 years with hemiplegia of at least three months' duration, who were able to ambulate a minimum distance of 16 (with or without an assistive device), and who had intact auditory and visual functions were included. The sample size was calculated using WinPEPI Software, version 11.38 (Brixton Health, London), with a 95% confidence level, 80% power, and a 0.05 significance level, resulting in a total sample size of 30 participants.

After obtaining written informed consent and completing baseline assessments, participants were randomly allocated into two groups using a simple randomization (chit) method. Equal numbers of chits labeled Group A (conventional physiotherapy) and Group B (AOT) were placed in an opaque container and thoroughly mixed before selection. No stratified randomization was performed due to the relatively small sample size and the homogeneous nature of the study population recruited from a single clinical setting. Baseline demographic and clinical characteristics were comparable between the groups, indicating adequate homogeneity prior to intervention.

Outcome measures

Our study employed the footprint method as one of the assessment techniques to evaluate gait parameters. This method represents an economical yet effective approach for gait analysis that can be implemented with minimal resources. In our protocol, participants walked along a designated pathway where their foot impressions were captured using pressure-sensitive materials. This technique allowed us to collect valuable quantitative data regarding multiple gait characteristics, including stride length and step width, which were particularly relevant to our investigation of circumduction gait. The simplicity of the footprint method made it especially suitable for our clinical setting while still providing reliable measurements. Previous validation studies have demonstrated impressive reliability coefficients for this technique, with values ranging from 0.921 to 1.00, indicating excellent consistency both between different examiners and across repeated measurements by the same examiner [9,10]. This high level of reliability supported our confidence in using the footprint method as a primary assessment tool, particularly when combined with our motion analysis using Kinovea software (developed by Joan Charmant and Contributors, Kinovea Open Source Project, Bordeaux, France), which provided complementary data on circumduction angles and had a reliability of >0.98 [11].

To assess the effectiveness of AOT, Kinovea Software (version 0.9.5) was used to measure circumduction angles, while the footprint method was employed to evaluate stride length and step width. Circumduction angle was measured to assess the degree of lateral deviation of the affected lower limb during the swing phase of gait. A video recording of the participant’s gait was obtained in the frontal plane while walking along a marked walkway. The circumduction angle was calculated by drawing: 1) a straight reference line representing the normal line of progression, and 2) a line representing the maximal lateral deviation of the affected foot during the swing phase.

The angle formed between these two lines was measured using motion analysis software/goniometric analysis and recorded in degrees. Higher circumduction angles indicated greater abnormal circumductory gait deviation. Stride length and step width were assessed using the footprint method. Participants were asked to walk along a straight walkway after applying washable ink/color to the soles of their footwear, leaving footprints on a paper walkway. Stride length was measured as the linear distance between two successive heel prints of the same foot. The measurement was recorded in centimeters using a measuring tape. Step width was determined by measuring the perpendicular distance between the midpoints of the heel centers of the right and left footprints. Three trials were recorded, and the average value was taken for analysis. No specific step or gait cycle was selected to normalize gait speed; instead, participants were encouraged to maintain a consistent walking pace across all trials. This approach was adopted to capture the participants' natural gait performance and ensure ecological validity of the assessment.

The AOT videos were specifically developed for the study and presented in the local language (Marathi) to ensure optimal comprehension and participant engagement. The videos demonstrated a healthy individual performing gait-related activities from frontal and sagittal views. To facilitate motor learning, the task was presented in a structured manner, beginning with individual gait components such as weight shifting, hip and knee flexion during swing phase, ankle dorsiflexion, foot placement, and limb advancement. These components were subsequently integrated into the complete gait cycle, allowing participants to observe both isolated movement elements and the entire walking task.

Each session consisted of observing the videos, followed immediately by physically executing the observed movements. The videos emphasized key features required to reduce circumduction gait, including adequate hip and knee flexion, toe clearance during swing, symmetrical step length, and proper weight transfer to the affected limb. The observation period and practice duration were standardized across participants, and the same sequence of videos was used throughout the intervention period to maintain consistency and reproducibility of the treatment protocol.

The detailed intervention protocol, including the duration, progression, and components of AOT and conventional physiotherapy, is presented in Table 1. The intervention lasted for three weeks, with sessions conducted four times per week.

**Table 1:** Intervention protocol This structured approach, which combines observation and physical practice, aims to facilitate motor relearning in stroke patients. Each week builds progressively on the skills learned, ensuring continual improvement through engagement with the rehabilitation process AOT: action observation therapy; ROM: range of motion

Week	Intervention	Duration	Details
Week 1	AOT + conventional physiotherapy	40-55 minutes	AOT: observation of videos focused on hip-knee flexion and hip rotation (20-30 min), followed by 5 minutes relaxation and 10 minutes motor imagery. Conventional physiotherapy included stretching, active/passive ROM, weight-bearing, and coordination exercises (20-25 minutes)
Week 2	AOT + conventional physiotherapy	35-50 minutes	AOT focused on foot placement, gait mechanics, and movement sequencing during gait, followed by relaxation. Conventional physiotherapy progressed to balance and coordination training with increased difficulty
Week 3	AOT + conventional physiotherapy	35-50 minutes	AOT emphasized complete gait cycle performance and refinement of movement technique. Conventional physiotherapy included functional gait activities on varied surfaces along with balance and coordination challenges

**Figure 1:**
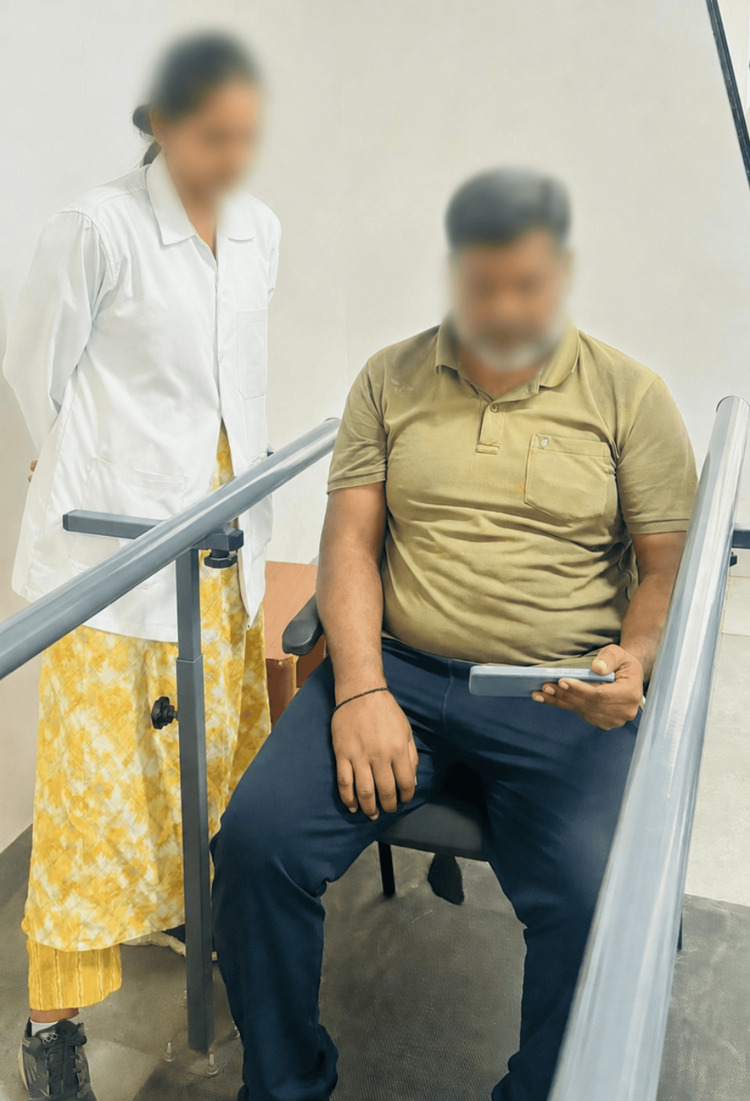
Observation of gait-related activities during action observation therapy

Figure 1 shows a stroke patient seated between parallel bars, attentively observing a gait-related task on a handheld device as part of AOT, while the therapist supervises to facilitate motor learning for gait training.

The data were entered into an Excel spreadsheet (Microsoft Corporation, Redmond, WA), tabulated, and subjected to statistical analysis. Various statistical measures, such as the mean, standard deviation, and significance tests, were used to analyze the data. The results were considered statistically significant if p < 0.05. Data were analyzed using GraphPad INSTAT (trial version 3.0632; Dotmatics, Boston, MA); a paired t-test was used to analyze the difference between pre- and postmeasurements within the group, and an unpaired t-test was performed to analyze the effectiveness of the AOT exercises when compared with conventional exercises on circumduction gait in hemiplegic patients.

## Results

The baseline demographic characteristics of participants in both groups were comparable, as shown in Table 2. The mean age of participants in Group A was 42.20 ± 5.17 years, while that of Group B was 42.13 ± 4.45 years. Each group consisted of 15 male and female participants, indicating a similar distribution of age and sex across the two groups prior to intervention.

**Table 2:** Baseline demographic characteristics of participants SD: standard deviation

Variable	Group A (n = 15)	Group B (n = 15)
Age (years), mean ± SD	42.20 ± 5.17	42.13 ± 4.45
Male, n (%)	13 (43.3%)	12 (40%)
Female, n (%)	2 (6.66%)	3 (10%)
Total participants	15	15

**Figure 2:**
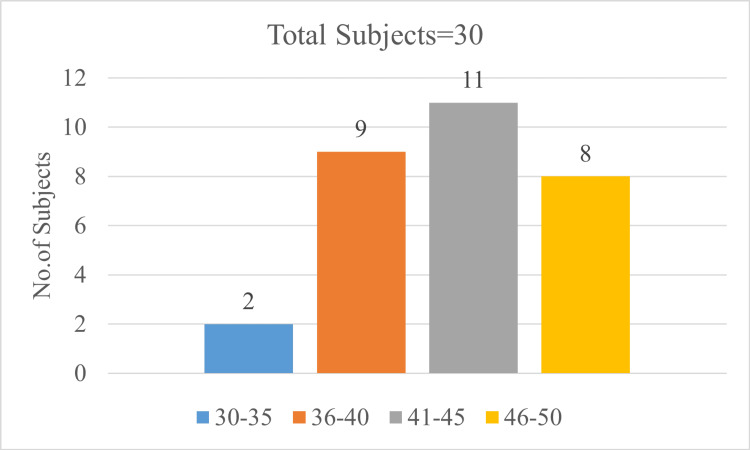
Age-wise distribution of subjects in the study

A total of 30 men and women of age group 30-50 years having circumduction gait volunteered to participate in the study, out of which five women (16.6%) and 25 men (83.3%) completed three weeks of the program. The detailed age-wise distribution of subjects included in the study is presented in Figure 2, which shows that the age group of 30-35 years is 6.6%,36-40 years is 30%, 41-45 years is 36.6%, and 46-50 years is 26.7%.

The pre- and postintervention comparison of circumduction angle, stride length, and step width for both Group A and Group B, along with their statistical analysis, is presented in Table 3.

**Table 3:** Within-group comparison of circumduction angle, stride length, and step width in Groups A and B Group A (conventional physiotherapy), n = 15; Group B (AOT), n = 15 Circumduction angle was measured in degrees (°), while stride length and step width were measured in centimeters Statistical analysis was performed using the paired t-test p < 0.05 was considered statistically significant SD: standard deviation; AOT: action observation therapy

Parameter	Group	Pre, mean ± SD	Post, mean ± SD	Mean difference	t value	p value	Result
Circumduction angle (in degrees)	Group A (conventional)	28.86 ± 4.98	24.26 ± 4.75	-4.6	2.58	0.0152	Significant
	Group B (AOT)	30.33 ± 5.47	23.8 ± 4.78	-6.53	17.36	<0.0001	Significant
Stride length (in cm)	Group A (conventional)	86.46 ± 3.37	91.73 ± 2.96	5.267	10.297	<0.0001	Significant
	Group B (AOT)	85.6 ± 3.29	109.6 ± 3.29	24	19.97	<0.0001	Significant
Step width (in cm)	Group A (conventional)	14.09 ± 1.54	13.34 ± 1.51	-0.746	11.297	<0.0001	Significant
	Group B (AOT)	18.82 ± 2.54	15.09 ± 1.86	-3.72	4.57	<0.0001	Significant

The between-group comparison of mean differences in circumduction angle, stride length, and step width following the intervention is presented in Table 4.

**Table 4:** Between-group comparison of mean change scores in circumduction angle, stride length, and step width following intervention Values are expressed as the mean ± SD of the change scores (difference between pretreatment and posttreatment values) Group A represents the conventional physiotherapy group (n = 15), and Group B represents the AOT Group (n = 15) Circumduction angle was measured in degrees (°), while stride length and step width were measured in centimeters Statistical analysis for between-group comparison was performed using the independent t-test p < 0.05 was considered statistically significant SD: standard deviation; AOT: action observation therapy

Parameter	Group	n	Mean	SD	Mean difference	t value	p value	Result
Circumduction angle (in degrees)	A	15	4.6	0.73	-1.93	4.58	<0.0001	Extremely significant
	B	15	6.53	1.45				
Stride length (in cm)	A	15	5.266	1.98	18.733	14.211	<0.0001	Extremely significant
	B	15	24	4.7				
Step width (in cm)	A	15	0.746	0.25	2.98	10.49	<0.0001	Extremely significant
	B	15	3.726	1.07				

## Discussion

This study examined the effectiveness of AOT as an adjunct to conventional physiotherapy in improving circumduction gait in stroke patients with hemiplegia. The results indicate that AOT, when combined with conventional therapy, significantly improves gait parameters, specifically the circumduction angle, stride length, and step width, compared with conventional therapy alone. These findings are consistent with the growing evidence supporting the role of the MNS in motor relearning and rehabilitation, suggesting a promising approach for stroke recovery [6].

The AOT group exhibited a significant reduction in the circumduction angle (mean difference: -6.53°, p < 0.0001) compared with the control group (mean difference: -4.6°, p = 0.0152). This suggests that AOT effectively targets the compensatory movement patterns associated with circumduction gait. This improvement may facilitate motor relearning while observing normal gait patterns, which likely encourages neural reorganization and strengthens cortical motor networks. Previous research, such as that by Mulder [12], supports this mechanism, indicating that action observation primes the motor system for subsequent execution, enhancing the efficiency of motor learning. The better outcomes in the AOT group highlight its potential to address motor control deficits more effectively than conventional methods alone.

Improvements in stride length further demonstrate AOT's impact, with the intervention group showing a significant increase (24 cm; p < 0.0001) compared with the control group (5.27 cm, p < 0.0001). This difference indicates that AOT reduces abnormal movements and promotes a more functional gait pattern, allowing patients to cover more ground with each step. Enhanced stride length is essential for improving walking efficiency and reducing energy expenditure, both of which are often compromised in circumduction gait. Similarly, the reduction in step width was more significant in the AOT group (3.72 cm, p < 0.0001) than in the control group (0.75 cm, p < 0.0001), indicating improved gait symmetry and stability. A narrower step width reflects better balance and coordination, thus reducing the risk of falls, a major concern for stroke survivors [4].

Several studies support these findings and reinforce AOT's role in stroke rehabilitation. For example, Ertelt et al. [13] conducted a randomized controlled trial on upper limb recovery in stroke patients, demonstrating that AOT significantly improved motor function compared with controls, with effects linked to mirror neuron activation. Similarly, Bang et al. [14] explored AOT's impact on gait in patients with chronic stroke, reporting improvements in walking speed and stride length following a four-week intervention. Park et al. [15] also found that AOT combined with treadmill training improved gait symmetry and reduced compensatory patterns in hemiplegic patients, closely aligning with this study's outcomes. Collectively, these studies validate AOT's efficacy in motor recovery, particularly for gait-related deficits, supporting its application in correcting circumduction gait.

Improvement of gait abnormalities through structured and progressive designs suggests that the AOT protocol may have successfully supported patients’ learning in a step-by-step manner (motor learning) by "chunking" gait components and progressing progressively by implementing the more complex movements of multijoint activities. Furthermore, video models that were culturally relevant to the patients provided an engaging and accessible means of providing support that helped support patients' efforts and ultimately improve their motivation throughout the four-week intervention; this is an often underemphasized factor that can influence rehabilitation outcomes.

The integration of AOT with conventional therapy likely enhances its effectiveness by combining observational learning with physical practice. Watching videos of normal gait, followed by active imitation, may reinforce neural pathways associated with proper movement execution, as suggested by Sarasso et al. [16]. The use of local language video scripts and a structured breakdown of gait components further tailored the intervention to the participants, potentially improving engagement and understanding. While conventional therapy resulted in some improvements in gait parameters, its lesser impact may be attributed to its focus on general strengthening and mobility rather than specific motor relearning [17].

These clinical findings suggest that AOT may be a cost-effective intervention in low-resource setting as AOT required only video playback for therapy and resulted in statistical improvement of gait measure/parameters. This is particularly significant in India where there has been a rising incidence of stroke but limited access to advanced rehabilitation. However, due to the use of equivalently lengthened conventional therapy in the control group, it is not yet clear if the improvements are due to an action observation or combined practice. More studies are needed to confirm these findings with larger sample sizes, longer follow-up timeframes, and additional comparison groups. Nevertheless, the results from this study were statistically significant (p < 0.05) and support the efficacy of AOT.

The study was limited by a short intervention duration, which may affect the generalizability of the findings. Long-term follow-up was not conducted to determine the sustained effects of AOT. Additionally, advanced gait analysis systems were not used, and measurements were primarily based on the footprint method and Kinovea software. Further studies with longer follow-up periods are recommended.

In conclusion, AOT represents a valuable addition to conventional stroke rehabilitation, effectively reducing circumduction gait and enhancing mobility. Its incorporation into clinical practice could improve functional independence and quality of life for stroke survivors, warranting further investigation and potential standardization in rehabilitation protocols.

## Conclusions

The purpose of this study was to evaluate the effectiveness of AOT in improving gait parameters in post-stroke individuals compared with conventional physiotherapy alone. The findings from the three-week pre-post experimental study demonstrated that participants who received AOT showed significant improvements in walking performance, indicating its potential as an effective adjunct to traditional rehabilitation approaches.

Statistical analysis revealed that AOT led to meaningful enhancements in key gait parameters, including a reduction in circumduction angle, an increase in total stride length, and a decrease in average step width. These improvements suggest better motor control, balance, and gait efficiency in the AOT group compared with those receiving only conventional physiotherapy, supporting the role of AOT in optimizing functional mobility after stroke.
